# Supplementary material for: Machine Learning Accelerates Crystallization for Structure Determination
Source: Angew Chem Int Ed Engl. 2026 May 4;65(25):e1218503. doi: 10.1002/anie.1218503 (PMC13266958; doi:10.1002/anie.1218503)

checkCIF/PLATON report

Structure factors have been supplied for datablock(s) lcz070

THIS REPORT IS FOR GUIDANCE ONLY. IF USED AS PART OF A REVIEW PROCEDURE FOR PUBLICATION, IT SHOULD NOT REPLACE THE EXPERTISE OF AN EXPERIENCED CRYSTALLOGRAPHIC REFEREE.

No syntax errors found. CIF dictionary Interpreting this report

Datablock: lcz070

Bond precision:	C-C = 0.0162 A		Wavelength=1.54184
Cell:	a=9.4870(1)	b=9.3552(1)	c=18.4803(1)
	alpha=90	beta=95.131(1)	gamma=90
Temperature:	100 K		

	Calculated	Reported
Volume	1633.61(3)	1633.61(3)
Space group	P c	P 1 c 1
Hall group	P -2yc	P -2yc
Moiety formula	C25 H15 Ag3 F18 N6 O2	C15 H3 Ag3 F18 N6, C10 H12 O2
Sum formula	C25 H15 Ag3 F18 N6 O2	C25 H15 Ag3 F18 N6 O2
Mr	1097.04	1097.04
Dx,g cm-3	2.230	2.230
Z	2	2
Mu (mm-1)	15.606	15.606
F000	1052.0	1052.0
F000’	1057.12	
h,k,lmax	12,11,23	12,11,22
Nref	6971[3491]	4527
Tmin,Tmax	0.069,0.082	0.468,1.000
Tmin’	0.011	

Correction method= # Reported T Limits: Tmin=0.468 Tmax=1.000
AbsCorr = MULTI-SCAN

```
Data completeness= 1.30/0.65        Theta(max)= 78.248

R(reflections)= 0.0376( 4499)       wR2(reflections)= 0.1066( 4527)

S = 1.070                  Npar= 488
```

The following ALERTS were generated. Each ALERT has the format
test-name_ALERT_alert-type_alert-level.
Click on the hyperlinks for more details of the test.

Alert level C

```
PLAT042_ALERT_1_C Calc. and Reported MoietyFormula Strings  Differ     Please Check
              Calc: C25 H15 Ag3 F18 N6 O2
              Rep.: C15 H3 Ag3 F18 N6, C10 H12 O2
PLAT090_ALERT_3_C Poor Data / Parameter Ratio (Zmax > 18) ........       6.87 Note
PLAT213_ALERT_2_C Atom F8               has ADP max/min Ratio .....       3.6 prolat
PLAT213_ALERT_2_C Atom F00L             has ADP max/min Ratio .....       3.3 prolat
PLAT213_ALERT_2_C Atom F017             has ADP max/min Ratio .....       3.8 prolat
PLAT213_ALERT_2_C Atom F01C             has ADP max/min Ratio .....       3.2 prolat
PLAT213_ALERT_2_C Atom F01F             has ADP max/min Ratio .....       3.3 prolat
PLAT220_ALERT_2_C NonSolvent   Resd 1  C   Ueq(max)/Ueq(min) Range        3.2 Ratio
PLAT220_ALERT_2_C NonSolvent   Resd 1  F   Ueq(max)/Ueq(min) Range        5.2 Ratio
PLAT234_ALERT_4_C Large Hirshfeld Difference F1       --C7        .      0.21 Ang.
PLAT234_ALERT_4_C Large Hirshfeld Difference F01C     --C00Z      .      0.17 Ang.
PLAT234_ALERT_4_C Large Hirshfeld Difference F01F     --C00Z      .      0.16 Ang.
PLAT342_ALERT_3_C Low Bond Precision on  C-C Bonds ..............    0.01625 Ang.
PLAT911_ALERT_3_C Missing FCF Refl Between Thmin & STh/L=    0.600         19 Report
                 2  2  0,  -3  6  1,  -1  0  2,   0  1  2,  -1  1  3,  -1  0  4,
                -1  1  4,   7  0  4,  -1  1  5,  -5  0  8,  -4  3  9,  -6  5 17,
                -3  5 19,  -1  0 20,  -2  0 22,  -2  1 22,  -1  0 22,  -1  1 22,
                 0  0 22,
PLAT918_ALERT_3_C Reflection(s) with I(obs) much Smaller I(calc) .          2 Check
PLAT934_ALERT_3_C Number of (Iobs-Icalc)/Sigma(W) > 10 Outliers ..          1 Check
                -1  2-10,
PLAT939_ALERT_3_C Large Value of Not (SHELXL) Weight Optimized S .      10.45 Check
PLAT971_ALERT_2_C Check Calcd Resid. Dens.  1.52Ang From F8            1.90 eA-3
PLAT977_ALERT_2_C Check Negative Difference Density on H00B      .      -0.39 eA-3
```

Alert level G

```
PLAT003_ALERT_2_G Number of Uiso or U(i,j)  Restrained non-H-Atoms          9 Report
PLAT004_ALERT_5_G Polymeric Structure Found with Maximum Dimension          1 Info
PLAT083_ALERT_2_G SHELXL Second Parameter in WGHT  Unusually Large       8.06 Why ?
PLAT143_ALERT_4_G s.u. on c - Axis Small or Missing .............    0.00010 Ang.
PLAT153_ALERT_1_G The s.u.'s on the Cell Axes   are Equal ..(Note)     0.0001 Ang.
PLAT186_ALERT_4_G The CIF-Embedded .res File Contains ISOR Records          2 Report
PLAT242_ALERT_2_G Low    'MainMol' Ueq as Compared to Neighbors of       C00T Check
PLAT242_ALERT_2_G Low    'MainMol' Ueq as Compared to Neighbors of         C7 Check
PLAT242_ALERT_2_G Low    'MainMol' Ueq as Compared to Neighbors of       C00Z Check
PLAT242_ALERT_2_G Low    'MainMol' Ueq as Compared to Neighbors of       C013 Check
PLAT242_ALERT_2_G Low    'MainMol' Ueq as Compared to Neighbors of       C01B Check
PLAT242_ALERT_2_G Low    'MainMol' Ueq as Compared to Neighbors of       C01E Check
PLAT434_ALERT_2_G Short Inter HL..HL Contact F1       ..F01C      .      2.81 Ang.
```

```
                                                       x,-1+y,z  =      1_545 Check
PLAT720_ALERT_4_G Number of Unusual/Non-Standard Labels ..........         65 Note
              Ag01    Ag02    Ag03    N004    O005    F006    N007    N008
              F009    O00A    N00B    F00C    F00D    N00E    F00F    N00G
              F00H    F00I    F00J    F00K    F00L    C00M    C00N    C00O
              H00O    F00P    C00Q    F00R    C00S    C00T    C00U    H00U
              C00V    C00W    H00A    H00B    C00X    H00X    C00Y    H00C
              H00D    C00Z    C010    H010    C011    H011    C012    H012
              C013    C014    H01A    H01B    C015    C016    F017    C018
              H01C    H01D    C019    C01A    H01E    C01B    F01C    C01E
              F01F
PLAT860_ALERT_3_G Number of Least-Squares Restraints ............         56 Note
PLAT883_ALERT_1_G Absent Datum for _atom_sites_solution_primary ..     Please Do !
PLAT910_ALERT_3_G Missing FCF Reflection(s) Below Theta(Min)[Deg]=       5.30 Note
                0  1  0,   1  0  0,   0  0  2,
PLAT912_ALERT_4_G Missing # of FCF Reflections Above STh/L=  0.600        108 Note
PLAT913_ALERT_3_G Missing # of Very Strong Reflections in FCF ....          1 Note
                0  0  2,
PLAT915_ALERT_3_G No Flack x Check Done: Low Friedel Pair Coverage         34 %
PLAT941_ALERT_3_G Average HKL Measurement Multiplicity ..........        2.6 Low
PLAT969_ALERT_5_G The 'Henn et al.' R-Factor-gap value ..........      4.866 Note
              Predicted wR2: Based on SigI**2  2.19 or SHELX Weight  9.97
PLAT978_ALERT_2_G Number C-C Bonds with Positive Residual Density.          0 Info
```

```
   0 ALERT level A = Most likely a serious problem - resolve or explain
   0 ALERT level B = A potentially serious problem, consider carefully
  19 ALERT level C = Check. Ensure it is not caused by an omission or oversight
  23 ALERT level G = General information/check it is not something unexpected

   3 ALERT type 1 CIF construction/syntax error, inconsistent or missing data
  19 ALERT type 2 Indicator that the structure model may be wrong or deficient
  11 ALERT type 3 Indicator that the structure quality may be low
   7 ALERT type 4 Improvement, methodology, query or suggestion
   2 ALERT type 5 Informative message, check
```

Validation response form

Please find below a validation response form (VRF) that can be filled in and pasted into your CIF.

```
# start Validation Reply Form
_vrf_PLAT042_lcz070
;
PROBLEM: Calc. and Reported MoietyFormula Strings  Differ     Please Check
RESPONSE: ...
;
_vrf_PLAT090_lcz070
;
PROBLEM: Poor Data / Parameter Ratio (Zmax > 18) ........       6.87 Note
RESPONSE: ...
;
_vrf_PLAT213_lcz070
;
PROBLEM: Atom F8              has ADP max/min Ratio .....        3.6 prolat
RESPONSE: ...
;
```

```
_vrf_PLAT220_lcz070
;
PROBLEM: NonSolvent   Resd 1  C   Ueq(max)/Ueq(min) Range        3.2 Ratio
RESPONSE: ...
;
_vrf_PLAT234_lcz070
;
PROBLEM: Large Hirshfeld Difference F1       --C7       .       0.21 Ang.
RESPONSE: ...
;
_vrf_PLAT342_lcz070
;
PROBLEM: Low Bond Precision on  C-C Bonds ..............    0.01625 Ang.
RESPONSE: ...
;
_vrf_PLAT911_lcz070
;
PROBLEM: Missing FCF Refl Between Thmin & STh/L=    0.600         19 Report
RESPONSE: ...
;
_vrf_PLAT918_lcz070
;
PROBLEM: Reflection(s) with I(obs) much Smaller I(calc) .          2 Check
RESPONSE: ...
;
_vrf_PLAT934_lcz070
;
PROBLEM: Number of (Iobs-Icalc)/Sigma(W) > 10 Outliers ..          1 Check
RESPONSE: ...
;
_vrf_PLAT939_lcz070
;
PROBLEM: Large Value of Not (SHELXL) Weight Optimized S .      10.45 Check
RESPONSE: ...
;
_vrf_PLAT971_lcz070
;
PROBLEM: Check Calcd Resid. Dens.  1.52Ang From F8              1.90 eA-3
RESPONSE: ...
;
_vrf_PLAT977_lcz070
;
PROBLEM: Check Negative Difference Density on H00B      .      -0.39 eA-3
RESPONSE: ...
;
# end Validation Reply Form
```

It is advisable to attempt to resolve as many as possible of the alerts in all categories. Often the minor alerts point to easily fixed oversights, errors and omissions in your CIF or refinement strategy, so attention to these fine details can be worthwhile. It is up to the individual to critically assess their own results and, if necessary, seek expert advice.

PLATON version of 17/09/2025; check.def file version of 17/09/2025

duplicate check

No duplication found

Datablock lcz070 - ellipsoid plot

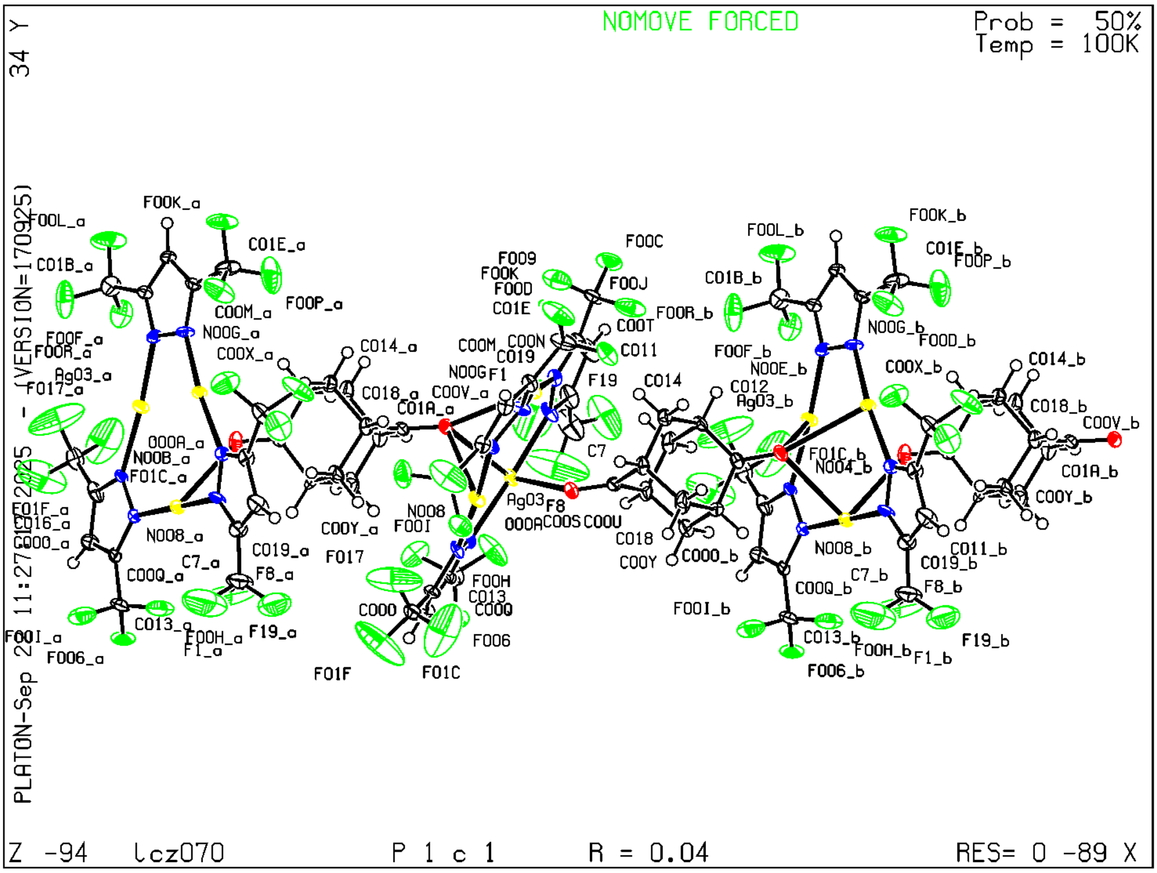

Supplement: Supplementary file 2 — Supporting File 2: anie72462‐sup‐0002‐DataFile.zip. [file ANIE-65-e1218503-s002.zip › supplementary_materials_crystallographic_data submit.zip!/cif_and_checkcif(1-114)/Ag3Pz3_70/Ag3Pz3_70_checkcif.pdf]
